# The efficacy and safety of *Jiedu Tongluo* granules for treating post-stroke depression with *qi* deficiency and blood stasis syndrome: study protocol for a randomized controlled trial

**DOI:** 10.1186/s13063-018-2633-4

**Published:** 2018-05-10

**Authors:** Ai-mei Zhao, Wen-ran Qiu, Li-jun Mao, Jun-guo Ren, Li Xu, Ming-jiang Yao, Kellie Bilinksi, Dennis Chang, Jian-xun Liu

**Affiliations:** 1 Graduate School, Beijing University of Chinese Medicine, Beijing, 100029 China; 2 Institute of Basic Medical Sciences, Xiyuan Hospital of China Academy of Chinese Medical Sciences, Haidian District, Beijing, 100091 People’s Republic of China; 3 Department of Neurology, Xiyuan Hospital of China Academy of Chinese Medical Sciences, Beijing, 100091 China; 4 NICM, Western Sydney University, Locked Bag 1797, Penrith, NSW 2751 Australia

**Keywords:** *Jiedu Tongluo* granules, Post-stroke depression, Randomized controlled trial, Chinese herbal medicine, *Qi* deficiency and blood stasis syndrome

## Abstract

**Background:**

Post-stroke depression (PSD) is the most common psychiatric complication after a stroke. The most frequently used antidepressants are selective serotonin receptor inhibitors (SSRIs) and serotonin and norepinephrine reuptake inhibitors (SNRIs), however, these exhibit a series of side effects. Traditional Chinese medicine has been used to treat PSD with few side effects. The aim of this study is to evaluate the efficacy and safety of *Jiedu Tongluo* granules for treating PSD with *qi deficiency* and *blood stasis syndrome*.

**Methods:**

The planned study is a double-blind, randomized, placebo-controlled pilot trial. Eighty participants will be randomly assigned to receive either treatment or placebo. The treatment group will receive *Jiedu Tongluo* granules (JDTLG) with conventional treatment, and the placebo group will receive placebo with conventional treatment for 8 weeks. The primary outcome is the effectiveness of JDTLG on depression after 8 weeks treatment, which is defined as a decrease of 50% or more in 17-item Hamilton Depression Scale (HAMD-17) score or clinical recovery (score < 7). Secondary outcomes are improvement in neurological function, degree of independence, activities of daily living, and TCM syndrome at each visit, which will be measured with National Institute of Health Stroke Scale (NIHSS), modified Rankin Scale (mRS), Barthel Index (BI) and TCM scale, respectively. Interleukin (IL)-6, IL-8, and small-molecule metabolites will be monitored to explore the mechanism of action of JDTLG on PSD. Safety measures include vital signs, results of electrocardiography, laboratory index (full blood count, kidney and liver function tests) and adverse events.

**Discussion:**

The purpose of this trial is to evaluate the therapeutic effects and safety of JDTLG in individuals with PSD with concomitant *qi deficiency* and *blood stasis syndrome*. If successful, the outcome of this trial will provide a viable treatment option for PSD patients.

**Trial registration:**

ClinicalTrials.gov ID: NCT03147053. Registered on 27 April 2017.

**Electronic supplementary material:**

The online version of this article (10.1186/s13063-018-2633-4) contains supplementary material, which is available to authorized users.

## Background

Post-stroke depression (PSD) is one of the most common complications of stroke and is characterized by depressed mood, generalized anxiety, and apathy [1–3]. The prevalence of PSD is approximately one third of stroke survivors at any one time [4], ranging from 39% to 52% within the first 5 years [5]. PSD patients experience poor functional outcomes [6] and increased mortality [7]. Several therapeutic strategies have been shown to be effective for PSD, including pharmacological, non-pharmacological interventions (e.g., psychotherapy, electroconvulsive therapy, and acupuncture), and combination therapies [8]. The most frequently studied agents are antidepressants, whereby the most thoroughly characterized agents are selective serotonin reuptake inhibitors (SSRIs) (e.g., fluoxetine, sertraline, citalopram) and serotonin and norepinephrine reuptake inhibitors (SNRIs) [9]. However, antidepressant medications exhibit a series of side effects, such as increased risk of hemorrhagic complications and stroke [10], although minor side effects such as nausea, diarrhea, fatigue, and dizziness are most commonly reported.

Traditional Chinese medicine (TCM) has been used to treat PSD and has demonstrated a more favorable safety profile than conventional medicine. A meta-analysis showed that *Liver-qi* regulation effectively ameliorates depressive symptoms, as well as enhancing quality of life among patients with PSD [11]. According to a data from a previous  research about the syndrome differentiation of ischemic stroke, the syndrome of *“qi deficiency* and *blood stasis”* might reach a proportion of 21.54% among the clinical patients, which would rank first within the different subtypes [12]. As a common complication of stroke, PSD can be divided into different syndromes, among which qi deficiency and blood stasis is an important subtype [13, 14]. *Jiedu Tongluo* granules (JDTLG) is a patented complex Chinese medicine formulation (No: 201510419571.3), which consist of seven herbs, including *Panax ginseng* (*Ren Shen*) 12.5 g/100 g, *Ginkgo biloba* (*Yin Xing Ye*) 25 g/100 g, *Hypericum perforatum L.* (*Guan Ye Lian Qiao*) 12.5 g/100 g, *Scutellaria baicalensis Georgi* (*Huang Qin*) 12.5 g/100 g, *Gardenia jasminoides Ellis* (*Zhi Zi*) 12.5 g/100 g, *Gastrodia elata Bl.* (*Tian Ma*) 12.5 g/100 g, and *Ligusticum chuanxiong hort* (*Chuan Xiong*) 12.5 g/100 g. According to the TCM theory, these herbs are believed to supplement *qi* and activate blood circulation, remove toxins and relieve stagnation, extinguish *wind*, and dredge collaterals. One previous study showed that JDTLG significantly improved depression-like behavior in animal stroke models [15]. *Panax ginseng* and *Ginkgo biloba*, key components of JDTLG, are known to possess neuro-protective effects [16–20]. Furthermore*, the antidepressant effects of Panax ginseng* [21] and *Ginkgo biloba*’s [22] might be derived from activation of the signaling pathway involving brain derived neurotrophic factor (BDNF). Our preclinical pharmacological experiments have shown that JDTLG has a neuroprotective and anti-apoptotic effect via up-regulating the expression of BDNF, hypoxia-inducible factor 1-alpha (HIF1α), cAMP-response element binding protein (CREB) and reducing the expression of vascular cell adhesion molecule1 (VCAM1) and matrix metallopeptidase 9 (MMP9). In addition, JDTLG can exert an anti-depressant function through increasing the expression of tropomyosin receptor kinase B (TrkB), mammalian target of rapamycin (mTOR), and 5-hydroxytryptamine (5-HT) receptor (unpublished data).

This study aims to explore whether JDTLG can improve depression and daily living compared with placebo in PSD participants with qi deficiency and blood stasis syndrome.

## Methods/design

### Study design

This is a double-blind, randomized, placebo-controlled, pilot trial designed to evaluate the safety and efficacy of JDTLG in treating PSD with qi deficiency and blood stasis syndrome. Eighty participants with PSD will be recruited from Xiyuan Hospital of China Academy of Chinese Medical Sciences. After obtaining written informed consent from participants or their legal guardian, participants will be randomly assigned to either the treatment or placebo group with a ratio of 1:1. Participants will undergo an 8-week treatment period and an 8-week follow up. The protocol design is based on the Consolidated Standards of Reporting Trials (CONSORT) guidelines and Standard Protocol Items: Recommendations for Interventional Trials (SPIRIT) Checklist (see Additional file 1).

### Recruitment

**Fig. 1:**
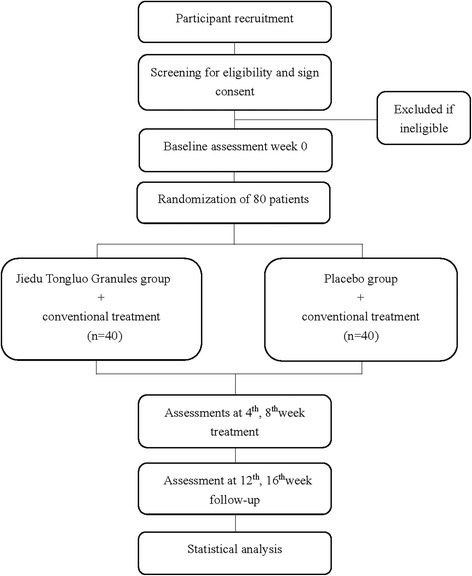
Study outline

Participants will be recruited by Internet advertisement and posters in the community and selected hospitals. Potential participants will be assessed by an attending trial physician who will determine whether the participant meets the eligibility criteria. The physician will explain the study’s aims, procedures, and possible side effects of participation in detail and provide the participant with a copy of the Participant Information Sheet. The recruitment started in April 2017 and is estimated to end in March 2018. If the potential participant is eligible and interested in participating, they will be invited for a baseline screening prior to randomization. An outline of procedures is illustrated in Fig. 1.

### Diagnostic criteria

A diagnosis of stroke will be established according to *all kinds of cerebrovascular disease diagnosis points* [23]. Clinical diagnosis of depression is based on the *Chinese Classification and Diagnostic Criteria of Mental Disorders-3* (CCMD-3) [24] and the *Diagnostic and Statistical Manual of Mental Disorders-IV* (DSM-IV). The TCM syndrome of qi deficiency and blood stasis in the study follows the *Guidelines for Clinical Research of New Chinese Medicine* [25]*.* The diagnostic standards are as follows:

1. Primary signs and symptoms include hemiplegia, tongue skew, tongue strong with no words, partial numbness or feeling diminished or disappeared, emotional depression or anxiety
2. Secondary signs and symptoms include dizziness, shortness of breath, dark lips, and pale tongue with petechial ecchymosis, string slide pulse

In the diagnosis of stroke involving both collateral and meridian, participants will be diagnosed with qi deficiency and blood stasis syndrome under condition of two or more primary signs/symptoms, as well as at least two primary signs/symptoms.

#### Inclusion criteria

1. Clinical diagnosis of stroke, showing symptoms of neurological deficit
2. Clinical diagnosis of depression according to the DSM-IV or CCMD-3
3. A score of 7–24 on the 17-item Hamilton Depression rating scale
4. Absence of psychiatric disease history or family history of psychosis before stroke
5. Conscious and cooperative, without aphasia or severe cognitive impairment 2 weeks after stroke
6. Hormones or psychotropic drugs not used within 1 month prior to enrollment
7. Normal liver and renal function tests
8. Aged 45–80 years old
9. Diagnosis of qi deficiency and blood stasis syndrome according to TCM pattern diagnosis
10. Able to provide voluntary signed informed consent

#### Exclusion criteria

1. Suspected secondary stroke caused by brain tumor, brain trauma
2. Unstable vital signs, or presence of severe primary hepatic or renal insufficiency (defined as the alanine aminotransferase (ALT), or the aspartate aminotransferase (AST), the serum creatinine concentration value twice of the normal upper limit)
3. Presence of other chronic disorders (e.g., chronic alcoholism or drug abuse)
4. Poorly controlled diabetes (the preprandial blood glucose is twice the upper limit of normal)
5. Currently pregnant or breast-feeding
6. Allergy to the ingredients in JDTLG
7. Currently participating in another clinical trial

### Withdrawal and discontinuation

Participants are able to voluntarily withdraw at any time during the trial. Participants who fail to complete the study, regardless of time or reason, will be considered as dropouts. The last recorded data for these participants will be included in data analyses. Data obtained from participants who experience unrelated complications, such as substantial change in disease status, exacerbation of depression or commence medication that could potentially interfere with JDTLG, will be treated as invalid. Withdrawal due to adverse events will be distinguished from withdrawal due to insufficient response. All adverse effects will be analyzed at the endpoint of the trial regardless of whether they are deemed related to the trial medication or not. If a participant withdraws due to a serious adverse event, it will be reported in accordance with the reporting requirements. Where appropriate, data collected by these participants will be used in intention-to-treat analyses.

### Intervention

Participants will be allocated to receive JDTLG or placebo for 8 weeks in addition to their usual conventional treatment (e.g., antiplatelet, lipid-lowering, antihypertensive, or antidiabetic therapy). The JDTLG and placebo granules (3.9 g/sachet) will be taken orally with warm water twice a day for 8 weeks. Product will be provided by China Resources Sanjiu Medical and Pharmaceutical Co., Ltd., Shenzhen, China (Production batch number: 161001 W). Analyses have shown that the quality of JDTLG is consistent with the Chinese Medicine Standards of the State Food and Drug Administration (SFDA). Active granules contain 3.9 g of JDTLG each, which is equivalent to 24 g of crude medicine. Placebo granules are composed of 10% crude JDTLG and 90% starch and are of identical appearance and smell as the active granules. Participants will be instructed to refrain from all other Chinese herbal decoctions or medicines used to treat PSD during the study. Participants can withdraw from the study and choose modern medical treatments, such as SSRI antidepressants, when their depressive symptoms worsen. All of the unused granules will be asked to be returned to the trial team and reasons of not taking the medicine will be recorded on the CRF.

### Outcomes and measures

#### Primary outcome

The primary outcome is the effectiveness of JDTLG on reducing depression after 8 weeks of treatment. Efficacy defined as a decrease of 50% or more in the 17-item Hamilton Depression Scale (HAMD-17) score or clinical recovery (score < 7) [26].

#### Secondary outcomes

- Improvement in neurological function at each appointment throughout the trial. Efficacy will be determined by a reduction of ≥ 50% or the total score of 0–1 in the 15-item National Institute of Health Stroke Scale (NIHSS) score (scores range from 0 to 42, whereby higher scores indicate a more severe neurological deficit) [27]
- The improvement in independence at each appointment as measured by a modified Rankin Scale (mRS) at each appointment throughout the trial (scores on this scale range from 0 to 6, with higher scores indicating greater disability) [28]
- The proportion of participants with a Barthel Index (BI) score of ≥ 90 at each appointment throughout the trial. The BI is used to evaluate the activities of daily living, ranging from 0 to 100, whereby higher scores indicate increased independence [29]
- The improvement of TCM syndrome at each appointment as measured by the reduction (reduction = (before treatment score − after treatment score)/before treatment score × 100%) in the following scale: clinical cure: reduction > 75%, markedly effective: reduction 51–75%, effective: reduction 25– 50%, invalid: reduction < 25% [30]

#### Exploratory outcomes

Interleukin (IL)-6, IL-8, and small-molecule metabolites will be monitored to explore the mechanism of PSD at baseline and weeks 4 and 8. Serum inflammatory cytokines such as IL-6 [31] and IL-8 [32], which are shown to participate in the pathological processes of both stroke and depression and provide some prediction values, will be detected using enzyme-linked immunosorbent assay (ELISA) techniques.

#### Safety measures

Safety measures will include vital signs, results of electrocardiography, a complete blood count, kidney and liver function tests (include blood lipid panel: total cholesterol, triglycerides, high-and low-density lipoproteins). All adverse events will be followed from the time of randomization to up to 16 weeks after the end of intervention.

### Safety assessment

Safety evaluation will be based on the incidence of adverse events (AEs) including clinically significant changes identified during physical examinations, vital signs (temperature, blood pressure, breathing and heart rate), and standard clinical laboratory tests (complete blood count, liver and renal function tests). Participants will be asked to report any abnormal events occurring at any time during the trial to the investigators. All details of related and unexpected AEs will be recorded on Case Report Forms (CRF). Safety will be monitored every 4 weeks, and for up to 16 weeks after the end of intervention.

### Study visits and procedures

The visit schedule for all evaluations (baseline, primary outcomes, secondary outcomes, exploring outcomes and safety indicators) is shown in Table 1. Participants will be assessed by the same investigator at each assessment. Baseline measurements, demographic characteristics, and medical history will be collected at the first visit only. All concomitant medications will be recorded during the treatment period (i.e., baseline to 8 weeks), including the name, dosage, and course of these medications. Primary and secondary outcomes and safety indicators will be collected every 4 weeks from baseline until week 16.

**Table 1:**
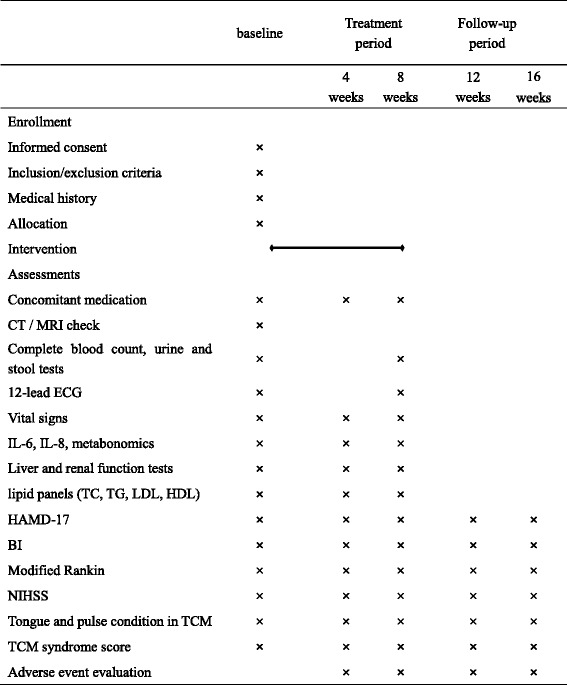
Measurement items and points of data capture

Abbreviations: CT Computed Tomography, MRI Magnetic Resonance Imaging, ECG electrocardiogram, IL interleukin, TC total cholesterol, TG triglycerides, LDL low density lipoprotein, HDL high density lipoprotein, HAMD-17 17-item Hamilton Depression Scale, BI Barthel Index, NIHSS National Institute of Health stroke scale, TCM traditional Chinese medicine.

### Randomization and blinding

A statistician from Xiyuan Hospital GCP Center will generate the randomization code. Eighty participants will be assigned to one of two groups at the ratio of 1:1, whereby the treatment and placebo groups will be coded A and B, respectively. The code will be placed in an envelope and subsequently sealed. The statistician will send the envelope directly to the product manufacturer for labeling of the active and placebo product according to the randomization code. All participants and attending physicians will be blinded to treatment assignment until study completion. Duplicated blinding codes will be given to the research institution and the manufacturer and will not be broken during the trial unless required to do so.

### Data management and quality control

The trial will be conducted according to the ICH Guidelines for Good Clinical Practice in effort to guarantee the rigor of the study. Data will be imported in the clinical data management system (CDMS) available at http://www.xyedc.com, and an independent organization will be responsible for the data management. The investigators are required to attend a series of training sessions which will cover correct administration of the assessments, participant instruction procedures regarding the use of study granules, collection of study variables, CRF completion, and use of the CDMS. Data is to be imported into the CDMS by two independent researchers within 1 week of collection. All discrepant entries will be marked and recorded for resolution at the clinical data system. After the first participant has completed, regular visits will be performed by an independent trial monitor who has been appointed by the trial sponsor. The monitor will review the two entries, resolve discrepancies, and recheck the CRFs and investigators’ source data if necessary.

### Compliance and retention

In order to maximize compliance and retention throughout the study, we will ensure the study schedule and requirements of participation are fully explained prior to obtaining consent, and that participants are aware of the potential risks and benefits of treatment. A copy of the signed informed consent form, containing the contact details of investigators, will be given to each participant so that they are able to contact the principal investigator if required. What is more, all participants will receive a telephone call before each follow-up appointment to remind them of the treatment time and location. Additionally, 8 weeks of medication will be given to participants in two separate sessions to avoid it being misplaced; i.e., at baseline and at week 4. We will try to prevent dropouts by providing ongoing support to participants. Transport allowance, free medical and relative examination will be provided for the participants.

### Sample size calculation

This is an exploratory clinical trial designed to provide scientific rationale and feasibility for clinical phase II pilot study and, therefore, is not fully powered. A sample size of 80 participants will be recruited, with 40 participants in each treatment group. Results from this study will be used to for sample size calculations in future studies.

### Statistical analysis

All of the data from this study will be analyzed by biostatisticians using SPSS 20.0 (IBM Corp., Armonk, NY, USA). Outcomes are based on the intention-to-treat analysis.

Baseline characteristics will be summarized by means of simple descriptive statistics and be compared between two study groups to assess covariate balance. The two-sample Student’s *t* test will be used for continuous variables and the chi-square test or Wilcoxon test will be used for categorical variables.

The primary analysis will be a comparison of the efficacy on depression between the two groups after 8 weeks of treatment. Pearson’s chi-squared test will be used to analyze the effective cases and logistic regression will be used to adjust for chance imbalances in the main prognostic variables between groups, such as age and gender.

Secondary analyses will include the improved cases in neurological function (NIHSS score reduction of ≥ 50% or the total score of 0–1), independence (mRS 0, 1, and 2), daily living (BI scores ≥ 90), and TCM syndrome. Secondary outcome analyses will be carried out according to standard statistical principles for comparison of parametric or non-parametric distributions as appropriate. Logistical regression analysis will be used to adjust for chance imbalances in the main prognostic variables between groups, such as age, NIHSS scores, BI scores, and TCM syndrome scores.

## Discussion

Depression is common after stroke and is related to with poor functional outcome and high mortality [33]. The association between depression and stroke has been established whereby stroke has been shown to increase the risk of PSD, and conversely, depression has been shown to be an independent risk factor for stroke [34]. The most commonly used antidepressants are SSRI and SNRIs, although they are not without side effects including increasing risk of hemorrhagic complications. As antidepressant medications have the potential to cause a series of adverse effects and are associated with poor patient compliance, it is important to explore other effective treatments that may have fewer side effects in the management of PSD. Chinese herbal medicine has the potential to offer an effective and safe treatment for PSD.

In this trial, we will investigate whether the JDTLG plus conventional treatment can reduce depression and improve daily living ability among participants who have recently suffered a stroke. We have previously shown that JDTLG effectively treats PSD in a rat model. This study will be conducted as a randomized, double-blind, placebo-controlled pilot crossover trial to provide evidence for the clinical efficacy of JDTLG.

The purpose of this prospective trial is to prove the therapeutic efficacy and safety of JDTLG. The outcome of the trial will provide evidence-based data regarding whether JDTLG is beneficial for PSD participants with qi deficiency and blood stasis syndrome. More importantly, the success of this study will support a large-scale clinical study to further consolidate the evidence for the use of JDTLG in PSD patients with qi deficiency and blood stasis syndrome.

This study has several limitations that require consideration. Firstly, as the study is being undertaken in one center; therefore, it may not necessarily be possible to extrapolate these results to other regions or ethnic groups. Secondly, the sample size is small and the treatment period short. However, this data will inform a subsequent multi-centered study compromising a sufficient sample size over a longer treatment period.

In conclusion, this prospective study will demonstrate that the JDTLG with conventional treatment is able to effectively and safely treat PSD with qi deficiency and blood stasis syndrome, thereby providing a new direction for treating PSD with minimal side effects.

### Trial status

The trial was initiated in April 2017 and is currently recruiting participants.

## Additional file

Additional file 1: SPIRIT Checklist. (PDF 211 kb)

## Abbreviations

AEs: Adverse events
BI: Barthel Index
CDMS: Clinical data management system
CRF: Case report form
CT: Computed tomography
ECG: Electrocardiogram
HAMD-17: 17-item Hamilton Depression Scale
IL: Interleukin
MRI: Magnetic resonance imaging
NIHSS: National Institute of Health Stroke Scale
PSD: Post-stroke depression
SNRI: Serotonin and norepinephrine reuptake inhibitors
SSRI: Selective serotonin reuptake inhibitors
TCM: Traditional Chinese medicine
